# Epidemiological characterization of *Mycobacterium caprae* strains isolated from wildlife in the Bieszczady Mountains, on the border of Southeast Poland

**DOI:** 10.1186/s12917-020-02581-3

**Published:** 2020-09-29

**Authors:** Blanka Orłowska, Monika Krajewska-Wędzina, Ewa Augustynowicz-Kopeć, Monika Kozińska, Sylwia Brzezińska, Anna Zabost, Anna Didkowska, Mirosław Welz, Stanisław Kaczor, Piotr Żmuda, Krzysztof Anusz

**Affiliations:** 1 Department of Food Hygiene and Public Health Protection, Institute of Veterinary Medicine, Warsaw University of Life Sciences (SGGW), Nowoursynowska 166, 02-787 Warsaw, Poland; 2 Department of Microbiology, National Veterinary Research Institute, Partyzantów 57, 24-100 Puławy, Poland; 3 Department of Microbiology, National Tuberculosis and Lung Diseases Research Institute (NTLD), Płocka 26, 01-138 Warsaw, Poland; 4 General Veterinary Inspectorate, Wspólna 30, 00-930 Warsaw, Poland; 5 County Veterinary Inspectorate, Młynarska 45, 38-500 Sanok, Poland; 6 University Centre of Veterinary Medicine UJ-UR, al. Mickiewicza 24/28, 30-059 Cracow, Poland

**Keywords:** Wild boar, Roe deer, European bison, Badger, *Mycobacterium caprae* genotyping, Spoligotyping, MIRU-VNTR, TB

## Abstract

**Background:**

The majority of animal tuberculosis (TB) cases reported in wildlife in Poland over the past 20 years have concerned the European bison inhabiting the Bieszczady Mountains in Southeast Poland: an area running along the border of Southeast Poland. As no TB cases have been reported in domestic animals in this region since 2005, any occurrence of TB in the free-living animals inhabiting this area might pose a real threat to local livestock and result in the loss of disease-free status. The aim of the study was to describe the occurrence of tuberculosis in the wildlife of the Bieszczady Mountains and determine the microbiological and molecular characteristics of any cultured strains. Lymph node samples were collected for analysis from 274 free-living animals, including European bison, red foxes, badgers, red deer, wild boar and roe deer between 2011 and 2017. Löwenstein–Jensen and Stonebrink media were used for culture. Molecular identification of strains was performed based on *hsp65* sequence analysis, the GenoType®MTBC (Hain Lifescience, Germany**)** test, spoligotyping and MIRU-VNTR analysis.

**Results:**

*Mycobacterium caprae* was isolated from the lymph nodes of 21 out of 55 wild boar (38.2%; CI 95%: 26.5%, 51.4%) and one roe deer. Since 2014, no new TB cases have been reported in the Bieszczady European bison population.

**Conclusions:**

The identification of TB in wild boar in the Bieszczady is an alarming phenomenon, which requires further investigation. The Bieszczady mountains are a precious, unique area, home to many protected species. However, it is also the only area in Poland where TB cases have been reported in free-living animals. The occurrence of TB in wild boar inhabiting this area might pose a real threat to local livestock and many of the protected species (for example European bison that can share feeding places with wild boar). Given this situation, ongoing monitoring of the prevalence of TB should be conducted, and protective measures should be considered.

## Background

Animal tuberculosis (TB) is a serious disease with potentially significant consequences for livestock farmers. However, while it is usually successfully detected in livestock with the use of tuberculin tests, it is challenging to identify TB-positive animals among wildlife.

Following the European Commission Decision 2009/342/EC of 23 April 2009, Poland was declared officially free of bovine tuberculosis. Even so, some animal tuberculosis (TB) cases (*Mycobacterium caprae* and *Mycobacterium bovis*) have been reported in wildlife in Poland over the past 20 years, with most of them concerning the European bison inhabiting the Bieszczady Mountains in Southeast Poland, a region bordered by Slovakia and Ukraine. The identification of disease outbreaks in this region resulted in the culling of two herds of European bison (*Bison bonasus caucasicus*), each counting more than 20 individuals: “Brzegi Dolne” (1997–2001) [1] and “Górny San” (2010–2013) [2, 3].

Another disturbing phenomenon in the Bieszczady Mountains region was the first confirmed case of TB caused by *Mycobacterium caprae* (*M.caprae*) in a wild boar (*Sus scrofa*) [4]: as no TB cases have been reported in domestic animals in the Bieszczady Mountains region since 2005, any occurrence of TB in free-living animals inhabiting this area might pose a real threat to local livestock and result in the loss of its disease-free status. In addition, its presence may also represent a potential source of infection for many of the other protected species inhabiting this area, such as the grey wolf, brown bear, lynx, wildcat or European bison [5]. The Bieszczady Mountains are one of the main habitat for free-living population of European bison (lowland-Caucasian breeding line of the European bison) in Poland. The main study area, is characterized by high forest coverage (70%) [6] and low livestock abundance: on average 5.2 cattle per 1 km^2^, 2 sheep per 1 km^2^, 0.25 goats per 1 km^2^ and 0.24 pigs per 1 km^2^ (unpublished data of the County Veterinary Inspectorate, Ustrzyki Dolne, Sanok). Of these animals, only cattle are included in the national TB eradication program, except when visible lesions suggestive of TB are identified in the animal. Unfortunately, the Bieszczady Mountains region is difficult to reach due to its mountainous terrain, with its highest peak being Tarnica at 1346 m a.s.l., as well as its high forest coverage and low human population. Also, it is difficult to monitor tuberculosis in the wildlife inhabiting this area, as it hosts many protected species, and diagnostic culling requires the consent of the Minister of the Environment.

To better understand the threat posed by TB, the aim of the present study was to describe the occurrence of tuberculosis in the wildlife of the Bieszczady Mountains and to determine the microbiological and molecular characteristics of the cultured strains.

## Results

**Fig. 1:**
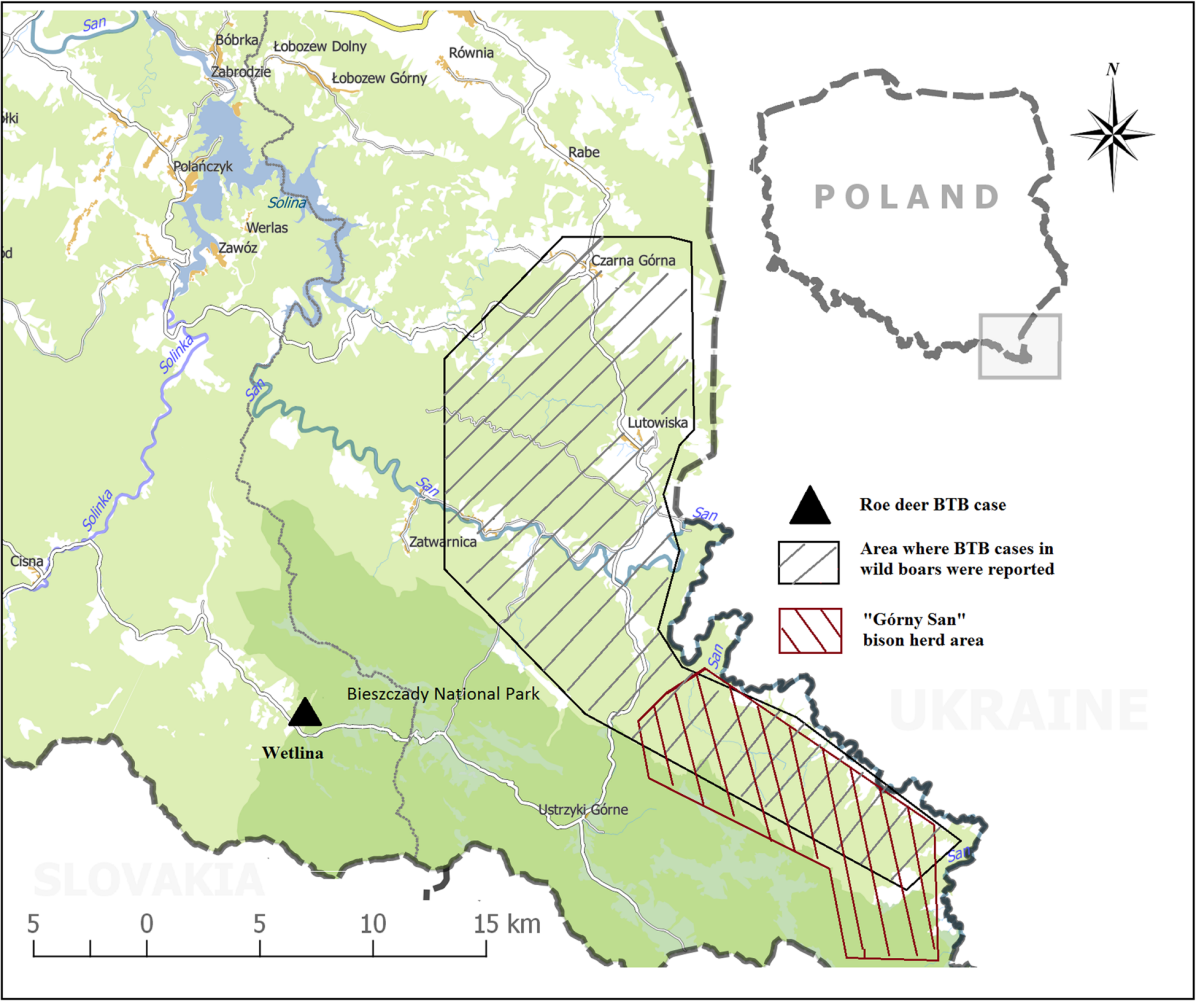
The area in which TB in wild boar were reported

A total of 274 free-living animals were sampled: 156 red foxes (*Vulpes vulpes*), 21 badgers (*Meles meles*), 13 red deer (*Cervus elaphus*), 55 wild boar (*Sus scrofa*), three roe deer (*Capreolus capreolus*) and 26 European bison (*Bison bonasus*). The microbiological analysis confirmed the presence of *Mycobacterium caprae* in the samples collected from 21 out of 55 wild boar (38.2%; CI 95%: 26.5%, 51.4%) in Bieszczady County and the single roe deer found near Wetlina (Cisna Commune, Lesko County) (Tables 1 and 2; Fig. 1). All 22 isolated strains of *M.caprae* shared an identical spoligotype 200,003,777,377,400 – SB2391 as assigned by www.Mbovis.org [7]. From this group of 22 strains, 20 shared a single MIRU-VNTR pattern (464,652,364,413,423), while the other two, isolated from two wild boar, (No.18 and 22; Table 1), had patterns that differed with regard to a single locus (464,552,364,413,423 and 463,652,364,413,423) (Table 1).

**Table 1:** Data about animals from which Mycobacterium caprae strains were isolated, and mycobacteria genotyping results

No.	Species/ study year	Sex	Age	Mycobacterium species	Spoligotype(as assigned by www.Mbovis.org)	MIRU
1	Roe deer /2013	M	7	*M.caprae*	SB2391	4 6 4 6 5 2 3 6 4 4 1 3 4 2 3
2	Wild boar /2013	M	2	*M.caprae*	SB2391	4 6 4 6 5 2 3 6 4 4 1 3 4 2 3
3	Wild boar /2013	F	2	*M.caprae*	SB2391	4 6 4 6 5 2 3 6 4 4 1 3 4 2 3
4	Wild boar /2013	F	2	*M.caprae*	SB2391	4 6 4 6 5 2 3 6 4 4 1 3 4 2 3
5	Wild boar /2013	F	1	*M.caprae*	SB2391	4 6 4 6 5 2 3 6 4 4 1 3 4 2 3
6	Wild boar /2013	F	1.5	*M.caprae*	SB2391	4 6 4 6 5 2 3 6 4 4 1 3 4 2 3
7	Wild boar /2014	F	1	*M.caprae*	SB2391	4 6 4 6 5 2 3 6 4 4 1 3 4 2 3
8	Wild boar /2014	N/D	N/D	*M.caprae*	SB2391	4 6 4 6 5 2 3 6 4 4 1 3 4 2 3
9	Wild boar /2014	N/D	N/D	*M.caprae*	SB2391	4 6 4 6 5 2 3 6 4 4 1 3 4 2 3
10	Wild boar /2014	N/D	N/D	*M.caprae*	SB2391	4 6 4 6 5 2 3 6 4 4 1 3 4 2 3
11	Wild boar /2014	N/D	N/D	*M.caprae*	SB2391	4 6 4 6 5 2 3 6 4 4 1 3 4 2 3
12	Wild boar /2017	M	1	*M.caprae*	SB2391	4 6 4 6 5 2 3 6 4 4 1 3 4 2 3
13	Wild boar /2017	F	3	*M.caprae*	SB2391	4 6 4 6 5 2 3 6 4 4 1 3 4 2 3
14	Wild boar /2017	M	2.5	*M.caprae*	SB2391	4 6 4 6 5 2 3 6 4 4 1 3 4 2 3
15	Wild boar /2017	F	1	*M.caprae*	SB2391	4 6 4 6 5 2 3 6 4 4 1 3 4 2 3
16	Wild boar /2017	N/D	N/D	*M.caprae*	SB2391	4 6 4 6 5 2 3 6 4 4 1 3 4 2 3
17	Wild boar /2017	M	2	*M.caprae*	SB2391	4 6 4 6 5 2 3 6 4 4 1 3 4 2 3
18	Wild boar /2017	N/D	N/D	*M.caprae*	SB2391	*4 6 4 5 5 2 3 6 4 4 1 3 4 2 3*
19	Wild boar /2017	M	2	*M.caprae*	SB2391	4 6 4 6 5 2 3 6 4 4 1 3 4 2 3
20	Wild boar /2017	M	0,5	*M.caprae*	SB2391	4 6 4 6 5 2 3 6 4 4 1 3 4 2 3
21	Wild boar /2017	N/D	0,5	*M.caprae*	SB2391	4 6 4 6 5 2 3 6 4 4 1 3 4 2 3
22	Wild boar /2017	F	N/D	*M.caprae*	SB2391	*4 6 3 6 5 2 3 6 4 4 1 3 4 2 3*

*M Male, F Female, N/D No data*

**Table 2:** Animals species from the Bieszczady region (Subcarpathian Voivodeship) investigated for tuberculosis in 2011–2017 and culture results

Species	Number of animals	
	investigated	from which *M.caprae* was isolated
Roe deer	3	1
Wild boar	55	21
Red deer	13	0
Badger	21	0
Red fox	156	0
European bison from the Bieszczady investigated after 2013	26	0

Twenty wild boar displayed typical TB lesions. Of these, nineteen (Nos 3 to 21, Table 1) demonstrated multiple yellowish tubercles measuring between 1 and 5 mm in their submandibular or in submandibular and mediastinal lymph nodes. One wild boar was heavily infected with lungworm (No.21, Table 1). One wild boar demonstrated typical TB lesions in submandibular, mediastinal, tracheobronchial lymph nodes and in lungs (multiple yellowish tubercles up to 1 cm) (No.22, Table 1). Another wild boar and the roe deer (Nos 1 and 2, Table 1) showed no anatomo-pathological lesions suggestive of TB.

## Discussion

The study conducted on samples collected from 274 free-living animals in the Bieszczady Mountains, viz. 26 European bison, 55 wild boar, 13 red deer, three roe deer, 21 badgers and 156 red foxes revealed TB infections in 21 wild boar from Bieszczady County and one roe deer from Wetlina in neighboring Lesko County. Before 2011, the only documented cases of tuberculosis in the Bieszczady were observed among some European bison, a badger and three grey wolves [1, 8, 9]. Since 2014, no new TB cases have been detected among European bison in the Bieszczady region; however, some have been reported in other areas of Poland [10]. It seems, therefore, that the action taken in 2012 to 2013, during which a herd of 24 European bison infected with *M.caprae* was culled, together with three individuals in an enclosure in Bukowiec, Bieszczady [9, 11–13] has prevented the disease from spreading among European bison in the Bieszczady, at least for now.

Although TB was also identified in one of the investigated roe deer, the small size of tested group, i.e. three animals, does not allow firm conclusions to be drawn about the prevalence of infection in this species. In contrast, one of our more significant findings is that TB was identified in 21 of the 55 tested wild boar. The first confirmed TB case in a wild boar in the Bieszczady region was reported in 2012 (a single individual) [4]; before this time, no cases of TB had been detected [8, 14]. The population density of wild boar was found to be 5–13 animals per 10 km^2^ of forest in the period 2011–2017 (Forest Management Plan of the Stuposiany and Lutowiska Forest Inspectorate, 2015; Unpublished data, Stuposiany Forest Inspectorate). In the present study, all the *M.caprae* strains isolated from the wild boar shared the same spoligotype, i.e. SB2391, and three very similar MIRU-VNTR patterns, which differed with regard to only one locus: 464,652,364,413,423, 464,552,364,413,423 and 463,652,364,413,423 (Table 1). It is believed that such differences in the number of repetitions at a single locus may indicate that the strains share a very close phylogenetic relationship, and that they may belong to a common transmission chain [15–17].

*M.caprae* strains isolated from the “Górny San” European bison herd, culled in 2011–2013, have the same spoligotype as strains isolated from boar (SB2391) and similar MIRU-VNTR patterns: 453,552,362,412,223, 453,652,362,412,223, 453,552,362,413,223, 423,552,342,411,223 and 423,552,362,412,223 [3]. To our knowledge, no other registered strains with such genetic patterns have been entered in the available databases, including those isolated from animals from other regions. The range of “Górny San” overlapped with the area from which the infected boar were obtained (Fig. 1). It is known that wild boar can share feeding places with European bison and can feed on bison carrion. Further studies are needed to determine the epidemiological link and the possible route of transmission of the source of infection.

Although tuberculosis has been reported in boar in many European countries, the identification of TB in wild boar in the Bieszczady is an alarming phenomenon, and one which should be investigated further. In certain environmental conditions, wild boar are known to act as reservoirs of bacteria from the *Mycobacterium tuberculosis* complex (MTBC) in the environment [18–20]. *M.caprae* infected wild boar may be a high-risk source of infection for other animals [21].

Surprisingly, no bacteria from the MTBC were isolated from the examined foxes, badgers and red deer. As the red fox is a very effective scavenger [22, 23] a carcass of a bison or boar infected with mycobacteria from the MTBC may represent a source of infection, and the red fox may be a spillover host of tuberculosis. In fact, bacteria from the MTBC have previously been isolated from this species [24, 25]; however, this research only studied the mandibular and mesenteric lymph nodes from red foxes. Although the mandibular and mesenteric lymph nodes are popular sources of study in TB research on predators, being the most common locations of lesions caused by TB infection, and mycobacteria are most often isolated from them [26, 27], subsequent studies should also include other lymph nodes, to increase their sensitivity. Together with the red fox, the Bieszczady region is also inhabited by larger predators and scavengers, such as the grey wolf, the brown bear and lynx. Competition between these species may significantly influence the possibility of red foxes being infected by mycobacteria from the MTBC from a carcass. The red fox, the smallest of the said predators, remains “the last to dine”. The grey wolf eats most of its prey [28] and usually begins with the viscera [29]. At the same time, in deer and boar, tuberculous lesions are often found in the lymph nodes of the head, in the chest and the abdomen [30–32]. It is also possible that foxes, when they feed on carcasses, are not left with much, because the infected tissues have been eaten by the larger predators. It should be noted that *M.caprae* have been isolated from wolves in the Bieszczady [5].

Badgers are easily infected with mycobacteria from the MTBC and hence their populations may also act as a tuberculosis reservoir [26, 33–35]. In the Bieszczady, tuberculosis mycobacteria have so far been isolated from only one individual [8] and the number of investigated badgers from the Bieszczady County may be too low to allow for conclusions to be drawn about the occurrence of tuberculosis in this population. It should be pointed out, however, that the density of population of badgers in the Bieszczady is low, with the density of badger setts in continental Eurasia estimated as 1.7 sett / 10 km^2^ [36]. Studies on badgers in the West Carpathians (Silesian Beskids) have found the average family group size to be 2.3 individuals and the population density as 2.2 individuals / 10 km^2^. The average density in the Carpathians, of which the Bieszczady are a part, is suspected to be similar [37].

*M.caprae* has been isolated on a number of occasions from red deer, indicating that it may act as a maintenance host for TB [38–42]. Although the population density of red deer was found to be 18–23 animals per 10 km^2^ of forest in the period 2011 to 2017, (Forest Management Plan of the Stuposiany and Lutowiska Forest Inspectorate, 2015; Unpublished data, Stuposiany Forest Inspectorate), few deer in this population have been studied for the presence of TB. The Bieszczady Mountains are also inhabited by a number of predatory species, such as wolves and bears. In this area, wolves prey mainly upon red deer, constituting over 70% of their consumed biomass, followed by European roe deer and wild boar [43, 44]. It would be worth investigating whether wolves play a regulatory role for populations if wild ungulates affected by TB in this area [5].

The high forest coverage, mountainous terrain, low livestock, low abundance of badgers and the coexistence of wild ungulate and predatory species in the region may also affect the circulation of MTBC in the studied environment [45].

Our study is limited by the relatively small number of animal samples tested, and hence our findings require further confirmation. As no established program to control TB in free-living animals currently exists in Poland, the material examined in the present study was obtained through the cooperation and goodwill of various other sources, including veterinarians, employees of forest inspectorates and biologists.

## Conclusions

The study conducted on samples collected from 274 free-living animals in the Bieszczady Mountains, revealed TB infections in 21 wild boar and one roe deer. The identification of TB in wild boar in the Bieszczady is an alarming phenomenon, and further studies are needed to determine the possible route of transmission and the source of infection. The occurrence of TB in wild boar inhabiting this area might pose a real threat to local livestock and many of the protected species. The Bieszczady mountains are a precious, unique area. However, it is also the only area in Poland where TB cases have been reported in free-living animals: the disease has so far been diagnosed in European bison, boar, grey wolves, as well as one roe deer and one badger [1–5, 8]. Given this situation, ongoing monitoring of the prevalence of animal tuberculosis should be conducted, and protective measures, such as the distribution of a TB vaccine, should be considered [46–48].

## Methods

### Sample collection

Samples were collected from a total of 274 free-living animals between 2011 and 2017: 156 red foxes (*Vulpes vulpes*), 21 badgers (*Meles meles*), 13 red deer (*Cervus elaphus*), 55 wild boar (*Sus scrofa*), three roe deer (*Capreolus capreolus*) and 26 European bison (*Bison bonasus*). These were subjected to microbiological testing to detect TB.

All investigated animals inhabited the Bieszczady Mountains region and their neighboring areas. The samples taken from the 156 foxes and four of the badgers were delivered by hunters to the Veterinary Hygiene Institution (VHI) in Krosno as part of the procedure for monitoring rabies and anti-rabies vaccination efficacy in red foxes. To qualify for use in mycobacterial tests, the samples had to be free from the rabies virus.

One of the roe deer, obtained from Wetlina (Cisna Commune), was a victim of a road accident (No. 1, Table 1). The European bison samples were taken from animals that had been found dead or had been culled for sanitary reasons with the consent of the Minister of Environment: the European bison samples were taken after 2013, after the elimination of the “Górny San” European bison herd.

The rest of the investigated animals had been hunted during the game season and were delivered by hunters for the study. All autopsies, age estimations and collection of research samples were conducted by veterinarians from the VHI in Krosno or by the County Veterinarian. Most of the investigated animals were adult individuals aged one to seven years. The samples taken from 156 foxes and four badgers by the VHI in Krosno comprised only mandibular and mesenteric lymph nodes. Among the remaining animals, all available lymph nodes were collected: the mandibular, retropharyngeal, mediastinal, tracheobronchial and mesenteric lymph nodes from all animals, together with the hepatic lymph nodes from the badgers.

All lymph nodes were subjected to thorough macroscopic examination. Lymph nodes with visible lesions were cultivated separately for the purposes of TB testing. The lymph nodes not displaying visible lesions collected from a given individual were pooled.

### Culture and molecular analysis

#### Isolation of mycobacteria

The collected samples were subjected to conventional procedures used for isolating mycobacteria. Briefly, the tissue samples were cleared of fat and cut into small fragments. The material was then ground and homogenized in 5% oxalic acid, with the whole being incubated for 15 minutes at 37 °C. After this time, the sample was centrifuged for 10 minutes at 3000–4000 rpm. The supernatant was removed, and the sediment flushed twice in sterile 0.9% sodium chloride. The sediment was then inoculated onto Löwenstein–Jensen and Stonebrink media (Oxoid, Germany) and incubated at 37 °C for 12 weeks.

#### Molecular analysis

The initial molecular identification of the strains was based on analysis of the *hsp65* sequence [49]. The investigated mycobacterium species was identified using the PRASITE database [50]. As this method does not differentiate species within the *Mycobacterium tuberculosis* complex (MTBC), further identification of MTBC strains was then performed using the GenoType MTBC (Hain Lifescience, Germany**)** molecular test, combined with spoligotyping and mycobacterial interspersed repetitive units-variable number tandem repeats (MIRU-VNTR) analysis.

The GenoType MTBC assay was performed in accordance with the manufacturer’s instructions. Spoligotyping was performed as described previously by Kamerbeek et al. (1997) [51]. To allow comparison with other databases, spoligotype patterns were entered in binary format into the SpolDB4 proprietary database of the Pasteur Institute of Guadeloupe [52]. Spoligotypes were also assigned according to international spoligotype nomenclature [7]. The MIRU-VNTR typing was performed using a public protocol [15]. The MIRU-VNTR-type was defined after combining the results for the 15 loci in the following order: MIRU 4, MIRU 10, MIRU 16, MIRU 26, MIRU 31, MIRU 40, VNTR 424, VNTR 577, VNTR 2165, VNTR 2401, VNTR 3690, VNTR 4156, VNTR 2163b, VNTR 1955 and VNTR 4052.

#### Statistical analysis

The 95% confidence interval (CI 95%) was calculated using Wilson score method.

The authors confirm that the ethical policies of the journal, as noted on the journal’s author guidelines page, have been adhered with accordance to DIRECTIVE 2010/63/EU OF THE EUROPEAN PARLIAMENT AND OF THE COUNCIL of 22 September 2010 on the protection of animals used for scientific purposes. All samples were collected post-mortem. According to competent authorities, this kind of research does not require ethics approval with respect to Polish law.

## Abbreviations

TB: Animal tuberculosis
*hsp65*: Heat-shock protein 65 gene
MIRU-VNTR: Mycobacterial interspersed repetitive units-variable number tandem repeats
*M.caprae*: *Mycobacterium caprae*
VHI: Veterinary Hygiene Institution
rpm: Revolutions per minute
MTBC: *Mycobacterium tuberculosis complex*

## Availability of data and materials

The datasets analyzed during the current study are available in the SpolDB4, SITVITWEB and Mycobacterium bovis spoligotype databases [http://www.pasteur-guadeloupe.fr:8081/SITVIT2/, http://www.pasteur-guadeloupe.fr/tb/spoldb4/spoldb4.pdf, https://www.mbovis.org/database.php].
